# Oxidative and nitrative stress as a pathogenic factor in multiple sclerosis

**DOI:** 10.1186/1753-6561-7-S1-P2

**Published:** 2013-01-30

**Authors:** Štefan Lukáč, Jana Muchová, Terézia Kalnovičová, Martina Horvathova

**Affiliations:** 1 Institute of medical chemistry, biochemistry and clinical biochemistry, Medical Faculty of Comenius University, Sasinkova 2, 813 72 Bratislava, Slovakia; 2 1st neurological clinic of Medical Faculty and University Hospital, Comenius University, Mickiewiczova 13, 813 69 Bratislava, Slovakia

## Background

Multiple sclerosis (MS) is an autoimmune disease of central nervous system, which unknown etiology, but recent studies suggest important role of oxidative stress in its pathogenesis. The aim of our study was to analyze various markers of oxidative and nitrative damage, their mutual correlations and correlations with the state of the blood-brain barrier (BBB) in multiple sclerosis patients. We also monitored the level of uric acid, an antioxidant.

## Methods

58 samples of blood plasma from patients with suspect MS and 43 ones from healthy people were analyzed. The function of BBB in tested group was evaluated using the QA index that indicated its damage in 7 males and 3 females. We estimated total antioxidant status, level of lipoperoxides, markers of protein deterioration by oxidative stress- protein carbonyls and by nitrative stress- 3-nitrotyrosine. Also uric acid concentration in males and females was detected. Student T-test and Pearson correlation coefficient were used for evaluating of statistical significance. Results are presented as average value ± SD. Statistical significance is calculated to the control group.

## Results

Summary of the results is in Table 1.

**Table 1:** 

PARAMETER	MS patients	Control group	p
**number**	58	43	
**age [years]**	36,52 ± 10,56	39,22 ± 14,88	p>0,1
**TAS[mmol/l]**	1,41 ± 0,47	1,91 ± 0,74	p<0,001
**lipoperoxidation **	79,17 ± 50,70	46,62 ± 27,36	p<0,001
**3-nitrotyrosine [nmol/l]**	104,51 ± 38,43	21,57 ± 3,67	p<0,001
**Protein carbonyls [nmol/mg P]**	0,44 ± 0,08	0,31 ± 0,01	p<0,001
**UA males [µmol/l]**	380,41 ± 86,57	320 ± 101,6	physiological
**UA females [µmol/l]**	298,86 ± 56,11	240 ± 101,6	physiological

Our study confirms earlier findings of decreased total antioxidative status in patients with MS and also increased lipoperoxidation, which positively correlated with the state of BBB. This finding induces role of lipid peroxidation by deterioration in the quality of blood-brain barrier. Elevated levels of protein carbonyls confirmed oxidative damage of plasma proteins, which are also attacked by nitrative stress, as evidenced by increased level of 3-nitrotyrosine and a positive correlation between 3-nitrotyrosine and protein carbonyls. Uric acid, which level was physiological, negatively correlated with protein carbonyls, what suggests its role in protection of proteins against oxidative stress, confirmed by the positive correlation with TAS.

## Conclusions

Based on these results, we can conclude that oxidative and nitrative stress are important factors in the pathogenesis of MS with effect on a wide range of substances. Therefore, it is necessary to pay attention to their reduction in the therapeutic process.

